# Pre-harvest bunch bagging as an eco-safe intervention for premium quality litchi production: insights from a multi-location study in India

**DOI:** 10.3389/fpls.2026.1779633

**Published:** 2026-02-25

**Authors:** Sunil Kumar, Evening Stone Marboh, Sushil Kumar Purbey, Mahesh Kumar Dhakar, Chandan Suravi Maiti, Ganeshi Lal Sharma, Vikas Kumar Sharma, Ashok Kumar Singh, Satish Chand, Samik Sengupta, Kalyan Chakraborti, Sumanjit Kaur, Nav Prem Singh, Sandeep Singh, Prakash Patil, S. Priya Devi, Rohit Kumar, Ashok Dhakad, Ipsita Samal, Bhagya Vijayan, Bikash Das

**Affiliations:** 1 Indian Council of Agricultural Research (ICAR)-National Research Centre on Litchi, Muzaffarpur, Bihar, India; 2 Regional Research Centre for Citrus, Indian Council of Agricultural Research (ICAR)-Central Citrus Research Institute, Bishwanath, Assam, India; 3 Indian Council of Agricultural Research (ICAR)-Mahatma Gandhi Integrated Farming Research Institute, Motihari, Bihar, India; 4 Farming System Research Centre for Hill and Plateau Region, Indian Council of Agricultural Research (ICAR)-Research Complex for Eastern Region, Ranchi, Jharkhand, India; 5 Nagaland University, Medziphema, Nagaland, India; 6 Indira Gandhi Krishi Vishwavidyalaya, Raipur, Chhattisgarh, India; 7 Dr. Yashwant Singh Parmar University of Horticulture and Forestry, Solan, Himachal Pradesh, India; 8 Govind Ballabh Pant University of Agriculture and Technology, Pantnagar, Uttarakhand, India; 9 Bihar Agricultural University, Bhagalpur, Bihar, India; 10 Bidhan Chandra Krishi Viswavidyalaya, Mohanpur, West Bengal, India; 11 Punjab Agricultural University, Ludhiana, Punjab, India; 12 Indian Council of Agricultural Research (ICAR)-Indian Institute of Horticultural Research, Bengaluru, Karnataka, India

**Keywords:** borer infestation, export quality, fruit cracking, non-woven polypropylene bags, sun burning

## Abstract

**Introduction:**

Pre-harvest fruit bagging is recognized as an eco-safe strategy to improve fruit quality and reduce biotic and abiotic stresses in high-value horticultural crops. However, its effectiveness in litchi (*Litchi chinensis* Sonn.) across diverse agro-climatic regions remains insufficiently documented. This study evaluated the impact of bagging materials and application timing on fruit damage, yield, and quality in litchi under multi-location conditions in India.

**Methods:**

Field experiments were conducted across nine major litchi-growing regions over four consecutive seasons (2020–2023). Seven treatments were tested in a factorial randomized block design, including white and pink non-woven polypropylene bags applied at 15, 25, and 30 days after fruit set (DAFS), along with an unbagged control. Data on fruit cracking, sunburn, borer infestation, yield, fruit weight, total soluble solids (TSS), anthocyanin content, and acidity were recorded. Statistical analyses included ANOVA, hierarchical clustering, and principal component analysis (PCA).

**Results:**

Bagging significantly reduced fruit cracking, sunburn, and borer infestation, with significant location × treatment interactions (P ≤ 0.05). Compared with the control, cracking and sunburn were reduced to ≤4–6% and ≤5–8%, respectively, while borer infestation remained below 3% in most locations. Yield increased by 10–35%, particularly when bagging was applied at 25–30 DAFS. Bagged fruits showed higher fruit weight, TSS, and anthocyanin content while maintaining desirable acidity.

**Discussion:**

Pre-harvest bagging is a robust and location-resilient practice that enhances litchi yield and marketable quality across diverse environments, supporting its adoption as a sustainable production strategy.

## Introduction

Litchi (*Litchi chinensis* Sonn.), an evergreen subtropical fruit crop belonging to the family Sapindaceae, is highly valued worldwide for its distinctive flavour, attractive red pericarp, and rich nutritional composition (Castillo-Olvera et al., 2025). India ranks second among the world’s leading litchi-producing countries, with cultivation extending over more than 100, 000 ha and an annual production of about 5.78 lakh metric tonnes (Anonymous, 2025). Traditionally, litchi cultivation in India has been concentrated in the eastern and sub-Himalayan belts of Bihar, West Bengal, Jharkhand and Uttarakhand (Nath et al., 2018). However, in recent years, cultivation has expanded into several non-traditional regions, including Punjab, Haryana, Himachal Pradesh, the North-Eastern states and parts of the southern plateau, driven by higher economic returns and opportunities for off-season production (Kumar et al., 2023).

Despite its rich nutritional value and high market demand, the commercial potential of litchi is often constrained by substantial pre-harvest and post-harvest losses associated with poor fruit colour development, fruit cracking, sun burn and insect pest infestation (Kumar et al., 2014a; Kumar et al, 2016; Marboh et al., 2017; Su et al., 2022). Further, the production is frequently challenged by abiotic stresses including irregular rainfall, sudden temperature variations and hailstorms, all of which severely reduce marketable yield and fruit quality (Lal et al., 2024, 2025). The prevailing production constraints often drive growers toward intensive and sometimes indiscriminate use of chemical pesticides, raising serious concerns related to food safety, environmental sustainability and human health. Furthermore, in recent years, export markets have shown a strong preference for residue-free, premium-quality fruits (Buthelezi et al., 2023; Georgoudaki et al., 2025). Consequently, there is a growing global emphasis on the adoption of good agricultural practices and eco-friendly technologies that reduce reliance on synthetic agrochemicals without compromising fruit quality and yield.

In this context, pre-harvest fruit bagging has emerged as a promising non-chemical intervention for improving fruit quality and reducing biotic and abiotic damage. The technique involves enclosing developing fruits or bunches in paper, fabric or polymeric bags, thereby modifying the fruit microenvironment and providing protection against excessive solar radiation, wind injury, temperature fluctuations and pest attack (Ali et al., 2021). Fruit bagging has been successfully adopted in several fruit crops such as peach (Jia et al., 2005), banana (Deepti et al., 2025), mango (Kumari et al., 2025; Nadeem et al., 2022), grape (Andreu-Coll et al., 2025; Luca et al., 2023), guava (Sharma et al., 2020; Srivastava et al., 2023), pear (Huang et al., 2009), sweet cherry (Feng et al., 2025), longan (Yang et al., 2009), pomegranate (Asrey et al., 2020; Habibli et al., 2025), and date palm (Alebidi et al., 2024), where it has been reported to improve external quality, enhance coloration and sweetness, and reduce pest and disease incidence (Ahmed et al., 2022; Frank, 2018). The practice is widely used commercially in countries such as Japan, China, Australia and the United States of America (Ali et al., 2021). Interestingly, several importing countries, including Mexico, Chile and Argentina, prefer or mandate the import of pre-bagged fruits to ensure superior quality and residue-free produce (Sharma et al., 2014).

With increasing domestic consumption and export demand and to strengthen India’s competitiveness in the global litchi market, the production of premium-quality litchi has gained significant commercial importance. Nevertheless, litchi in India is still largely produced without pre-harvest bagging for both domestic and export markets. Although recent studies have indicated that the techniques of bagging can enhance pericarp coloration, improve pulp-to-peel ratio and reduce pericarp browning (Pongener et al., 2025), most of the available investigations have been restricted to single locations or individual seasons. Consequently, information on the consistency and reliability of bagging responses under contrasting climatic conditions remains limited. Given the vast agro-climatic variability across India, ranging from humid subtropical and tropical to semi-arid and hill ecosystems, the physiological response of litchi fruit to different bagging materials is expected to differ substantially. Therefore, a coordinated multi-location evaluation is essential to understand the robustness, adaptability and scalability of this eco-safe intervention across regions.

The present investigation was undertaken under the aegis of the ICAR–All India Coordinated Research Project (AICRP) on Fruits to systematically evaluate the effect of different bagging materials and times on yield and fruit quality across nine major litchi-growing regions of India. This study represents the first comprehensive multi-location evaluation of litchi bunch bagging conducted in India and provides a scientific basis for developing region-specific, eco-safe recommendations for quality assurance in commercial litchi cultivation.

## Materials and methods

### Experimental sites and climatic conditions

**Figure 1:**
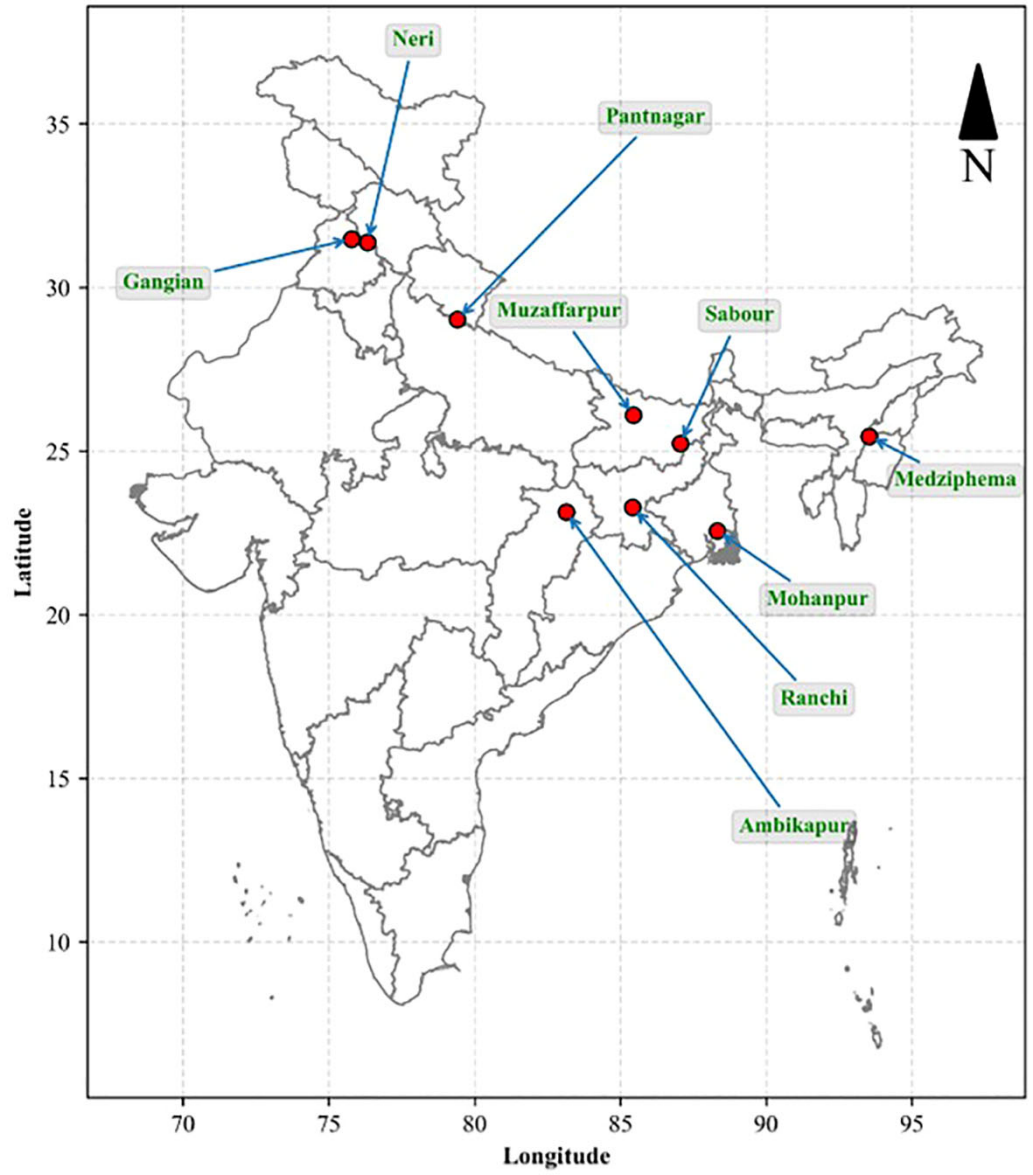
Geographical distribution of experimental sites of the multi-location study across major litchi-growing regions of India. The map was generated using Python based on the geographic coordinates (latitude and longitude) of each location.

The present multi-location study was conducted for four consecutive years (2020–2023) at nine centres representing the major litchi-growing agro-climatic regions of India under coordinated field conditions. The locations included Medziphema (Nagaland), Mohanpur (West Bengal), Sabour (Bihar), Muzaffarpur (Bihar), Ranchi (Jharkhand), Ambikapur (Chhattisgarh), Pantnagar (Uttarakhand), Neri (Himachal Pradesh), and Gangian (Punjab). The geographical distribution of the experimental sites, arranged from east to west across the country, is illustrated in **Figure 1**. A summary of the prevailing climatic characteristics of each experimental site is presented in **Table 1**. These data were used to characterize the agro-climatic variability across locations and to interpret the differential response of litchi fruit to bagging treatments under contrasting environmental conditions.

**Table 1:** Agro-climatic characteristics of the experimental centres and the cultivars used for the study.

Centre (State)	Agro-climatic zone	Climatic characteristics	Cultivar
Medziphema (Nagaland)	Humid subtropical hill region	High rainfall with a moderate thermal regime	*Shahi*
Mohanpur (West Bengal)	Humid tropical plain	High monsoonal rainfall and prolonged atmospheric humidity during fruit development	*Bombai*
Sabour (Bihar)	Humid subtropical alluvial plains	Hot summers with moderate to high relative humidity during fruit maturity	*Purbi*
Muzaffarpur (Bihar)	Humid subtropical alluvial plains	Hot summers with moderate to high relative humidity during fruit maturity	*Shahi*
Ranchi (Jharkhand)	Subtropical plateau region	Moderate temperature fluctuations with comparatively lower humidity than the plains	*Shahi*
Ambikapur (Chhattisgarh)	Tropical sub-humid region	Comparatively higher temperatures during fruit maturation	*Rose Scented*
Pantnagar (Uttarakhand)	Tarai humid subtropical region	High humidity with relatively cooler night temperatures	*Rose Scented*
Neri (Himachal Pradesh)	Mid-hill temperate region	Mild summers with relatively low atmospheric humidity	*Dehradun*
Gangian (Punjab)	Semi-arid irrigated plains	Hot, dry summers with relatively low relative humidity during fruit growth and maturity	*Dehradun*

### Plant material and experimental design

The experiment was conducted on locally important commercial litchi (*Litchi chinensis* Sonn.) cultivars at each participating centre, selected based on their regional adaptation and commercial popularity (**Table 1**). Uniform, healthy, and regularly bearing trees of comparable age (20–25 years) and vigor were selected at all centres to maintain experimental consistency. The experiment was laid out in a Randomized Block Design (RBD) with four replications at each location. Each treatment was imposed on four uniformly vigorous and regularly bearing trees, and twenty healthy and uniformly developed panicles per tree were selected for bagging according to the respective treatment schedules. Uniform cultural practices were followed across all experimental centres throughout the study period. No insecticide sprays were applied after bagging, and all plant protection measures were kept uniform for all treatments, except for the bagging interventions under investigation.

### Bagging materials and the time of application

The experiment comprised seven treatments based on the colour of non-woven polypropylene bags and the time of bagging (**Table 2**). UV-treated non-woven polypropylene bags (dimensions 20 × 16 inches, 30 GSM thickness, provided with drawstrings) of white and pink colours were used for bagging of litchi bunches. Bagging was carried out at 15, 25, and 30 days after fruit set, as per the treatment schedule. Uniform, healthy fruit bunches were gently inserted into the bags without causing mechanical injury to the developing fruits. Bags were securely tied at the basal end of the panicle using soft thread, ensuring adequate space for normal fruit growth and proper aeration. Bags were removed carefully at harvest to facilitate the recording of yield and fruit quality parameters.

**Table 2:** Details of bagging treatments applied in the experiment.

Treatment code	Colour	Time of bagging (DAFS)
T_1_	White colour non-woven polypropylene bag	15
T_2_	White colour non-woven polypropylene bag	25
T_3_	White colour non-woven polypropylene bag	30
T_4_	Pink colour non-woven polypropylene bag	15
T_5_	Pink colour non-woven polypropylene bag	25
T_6_	Pink colour non-woven polypropylene bag	30
T_7_ (Control)	—	Fruits left exposed to natural environmental conditions

DAFS, Days after fruit set.

### Harvesting procedure and measurement of cracking, sunburn and borer incidence

Fruits were harvested at commercial maturity based on the standard calendar dates followed at each experimental centre, corresponding to the regional harvesting season of litchi. Harvesting of all treatments at a given location was carried out on the same calendar date to avoid bias due to differences in maturity. Calendar-date–based harvesting was adopted to ensure practical uniformity and comparability under multi-location conditions, as fruit maturity in litchi is strongly influenced by regional climate, varietal differences, and orchard microenvironment. This approach minimized experimental bias arising from asynchronous flowering and fruit development across diverse agro-climatic zones.

The incidence of fruit cracking, sunburn and fruit borer infestation was recorded at the time of harvest. For each treatment, the total number of fruits harvested from the tagged panicles was counted, and the number of fruits affected by each disorder was recorded separately. Fruit cracking (%) was calculated as the proportion of cracked fruits to the total number of fruits per bunch at harvest. Sunburn incidence (%) was recorded as the percentage of fruits showing visible symptoms such as peel discoloration, hardening, or necrotic patches. The incidence of fruit borer (*Conopomorpha sinensis*) was determined by counting the number of infested fruits per bunch at harvest and expressing it as a percentage of the total fruits, based on the presence of entry holes, frass, and internal feeding damage.

### Assessment of yield and fruit quality parameters

Fruit yield is expressed as kilograms per tree (kg tree^-^¹) and was computed by weighing the total harvested fruits from each experimental tree using a digital balance (Model: DS-415 Series, Essae Teraoka Ltd., India). For fruit weight, twenty representative fruits were randomly sampled from each replication and weighed individually using an electronic balance, and mean fruit weight was expressed in grams (g). Anthocyanin content of litchi pericarp was estimated following the standard spectrophotometric method with minor modifications. Fresh, healthy fruits were collected at harvest, and the pericarp was separated immediately. Five grams of fresh pericarp tissue were finely macerated and extracted with acidified methanol (methanol: 1 N HCl, 85:15 v/v). The extraction was carried out in the dark at 4 ± 1 °C for 24 h to ensure complete dissolution of the pigments. The extract was filtered through Whatman No. 1 filter paper, and the absorbance of the clear filtrate was recorded at 530 nm and 657 nm using a UV–Visible spectrophotometer (Agilent Cary 60 UV-Vis, USA) against an appropriate reagent blank. The anthocyanin content was calculated using standard relationships and expressed as mg 100 g^-^¹ fresh weight (FW) of pericarp (Zhang et al., 2004). Total soluble solids (TSS) were determined from freshly extracted pulp using a digital hand refractometer (Atago, Tokyo, Japan) and expressed as °Brix at 20 °C. Titratable acidity of pulp was estimated using a standard acid–base titration method. Homogenized pulp extract was titrated against standard sodium hydroxide using phenolphthalein as an indicator, and acidity was expressed as percentage citric acid equivalent on a fresh weight basis. All measurements were done in triplicate.

### Statistical analysis

The experiment was conducted in a factorial randomized block design (FRBD) with location (nine levels) and bagging treatment (seven levels) as fixed factors. All treatments were replicated within each location, and observations were recorded on yield, fruit quality, and damage-related traits. Data were analyzed using two-factor analysis of variance (ANOVA) appropriate for a randomized block design to evaluate the main effects of location (L), treatment (T), and their interaction (L × T). The significance of effects was tested using F-tests at P ≤ 0.05, and treatment means were separated where significant differences were observed.

To assess overall treatment performance based on multiple traits, multivariate analyses were performed using R statistical software (v4.1.1). Treatment-wise means for fruit weight, total soluble solids, sunburn, fruit cracking, borer infestation, yield, anthocyanin content, and titratable acidity were used for analysis. Prior to multivariate analysis, all variables were standardized by Z-score transformation to account for differences in measurement scale. Hierarchical cluster analysis was conducted using Euclidean distance and Ward’s minimum variance method (Ward.D2), and results were visualized as a clustered heatmap. In addition, principal component analysis (PCA) was performed using the correlation matrix, retaining principal components with eigenvalues greater than one. PCA biplots were generated to examine relationships between treatments and traits and to identify treatments associated with higher yield and fruit quality attributes or increased incidence of physiological disorders and pest damage.

## Results

### Effect of pre-harvest bagging on fruit cracking, sunburn, and borer infestation

Fruit cracking, sunburn, and borer infestation were significantly influenced by location and bagging treatments, with a significant location × treatment interaction for cracking and borer infestation, while the interaction for sunburn was not significant (**Tables 3**–**5**). Across all locations, the unbagged control (T_7_) consistently recorded the highest incidence of fruit cracking, sunburn, and borer infestation, underscoring the vulnerability of exposed fruits to fluctuating temperature, solar radiation, and insect pressure. High cracking and sunburn were particularly evident at Ranchi, Ambikapur, Muzaffarpur, Pantnagar, and Gangian, whereas borer infestation under the control reached alarming levels at Muzaffarpur, Ranchi, Ambikapur, Mohanpur, and Neri. In contrast, all bagging treatments markedly reduced the incidence of physiological disorders and pest infestation. Fruit cracking and sunburn were generally restricted to below 5–7% under bagged treatments across locations, compared with substantially higher levels in the control. At Sabour and Mohanpur, bagging almost completely eliminated fruit cracking and sunburn, highlighting the effectiveness of microclimate moderation under humid conditions.

**Table 3:** Location-wise effect of bagging treatments on fruit cracking (%).

Treatment	Medziphema	Mohanpur	Sabour	Muzaffarpur	Ranchi	Ambikapur	Pantnagar	Neri	Gangian
T_1_	3.54 ± 1.59 ^hijkl^	0.68 ± 0.06 ^kl^	0.00 ± 0.00 ^l^	2.75 ± 0.40 ^ijkl^	6.20 ± 0.47 ^fghij^	3.37 ± 0.43 ^hijkl^	3.73 ± 0.13 ^ghijkl^	1.93 ± 1.93 ^fghijk^	5.43 ± 2.39 ^fghijk^
T_2_	2.54 ± 0.47 ^ijkl^	0.65 ± 0.05 ^kl^	0.00 ± 0.00 ^l^	3.44 ± 0.73 ^hijkl^	7.38 ± 0.91 ^efghij^	2.27 ± 0.28 ^ijkl^	5.57 ± 0.36 ^bcde^	2.00 ± 2.00 ^bcdef^	6.35 ± 2.42 ^fghij^
T_3_	2.74 ± 0.54 ^ijkl^	0.78 ± 0.07 ^kl^	0.00 ± 0.00 ^l^	3.88 ± 1.03 ^ghijkl^	8.57 ± 2.46 ^defghi^	1.49 ± 0.27 ^jkl^	4.65 ± 0.83 ^ghijkl^	4.50 ± 2.72 ^jkl^	9.09 ± 2.13 ^defghi^
T_4_	1.31 ± 0.22 ^jkl^	0.76 ± 0.06 ^kl^	0.00 ± 0.00 ^l^	3.14 ± 0.46 ^hijkl^	4.65 ± 0.37 ^ghijkl^	3.33 ± 0.11 ^hijkl^	4.02 ± 0.11 ^ghijkl^	1.83 ± 1.83 ^ijkl^	5.90 ± 1.07 ^fghijk^
T_5_	1.82 ± 0.47 ^jkl^	0.57 ± 0.04 ^kl^	0.00 ± 0.00 ^l^	4.00 ± 0.78 ^hijkl^	6.08 ± 0.44 ^fghij^	2.83 ± 0.16 ^ijkl^	5.01 ± 0.06 ^fghijk^	1.95 ± 1.95 ^ghijkl^	9.19 ± 2.00 ^defghi^
T_6_	2.05 ± 0.30 ^ijkl^	0.71 ± 0.06 ^kl^	0.00 ± 0.00 ^l^	3.85 ± 0.62 ^ghijkl^	6.81 ± 0.62 ^efghij^	1.55 ± 0.47 ^jkl^	5.03 ± 0.93 ^fghijk^	3.95 ± 2.28 ^jkl^	9.12 ± 1.22 ^defghi^
T_7_ (Control)	6.78 ± 2.37 ^efghij^	1.03 ± 0.13 ^jkl^	9.41 ± 0.23 ^l^	13.01 ± 0.28 ^ghijkl^	23.53 ± 0.42 ^a^	19.50 ± 0.68 ^ab^	12.17 ± 0.08 ^bcdef^	13.48 ± 3.74 ^jkl^	12.83 ± 2.95 ^bcdef^
F-value									
Location	35.85								
Treatment	68.66								
Location* Treatment	3.23								

Values ± S.E. within a column followed by different letters differ significantly at *P* ≤ 0.05.

**Table 4:** Location-wise effect of bagging treatments on sunburn (%).

Treatment	Medziphema	Mohanpur	Sabour	Muzaffarpur	Ranchi	Ambikapur	Pantnagar	Neri	Gangian
T_1_	5.83 ± 5.29 ^fghijkl^	0.94 ± 0.03 ^kl^	0.00 ± 0.00 ^l^	2.96 ± 0.54 ^hijkl^	4.52 ± 0.63 ^ghijkl^	1.97 ± 0.92 ^ijkl^	6.97 ± 0.69 ^efghijk^	5.57 ± 5.57 ^ghijkl^	7.99 ± 3.47 ^efghi^
T_2_	5.84 ± 4.15 ^fghijkl^	0.89 ± 0.02 ^kl^	0.00 ± 0.00 ^l^	4.97 ± 0.46 ^ghijkl^	6.63 ± 1.06 ^efghijkl^	6.80 ± 0.87 ^efghijkl^	7.96 ± 0.48 ^efghij^	5.07 ± 5.07 ^ghijkl^	4.25 ± 3.08 ^efghijk^
T_3_	5.01 ± 3.66 ^ghijkl^	1.00 ± 0.04 ^kl^	0.00 ± 0.00 ^l^	7.05 ± 0.36 ^efghijk^	7.22 ± 1.79 ^efghijk^	5.80 ± 1.36 ^fghijkl^	8.29 ± 0.67 ^efghi^	4.12 ± 4.12 ^hijkl^	13.27 ± 1.92 ^cdefg^
T_4_	1.20 ± 0.78 ^kl^	0.99 ± 0.03 ^kl^	0.00 ± 0.00 ^l^	3.74 ± 0.32 ^hijkl^	3.56 ± 0.36 ^hijkl^	2.81 ± 0.49 ^hijkl^	8.64 ± 0.63 ^efghi^	3.39 ± 3.39 ^hijkl^	8.92 ± 2.09 ^efghi^
T_5_	2.43 ± 1.52 ^ijkl^	0.86 ± 0.03 ^kl^	0.00 ± 0.00 ^l^	4.38 ± 0.31 ^ghijkl^	5.16 ± 0.48 ^ghijkl^	3.52 ± 0.16 ^hijkl^	7.91 ± 0.36 ^efghij^	5.19 ± 5.19 ^ghijkl^	7.40 ± 1.95 ^efghijk^
T_6_	3.10 ± 1.81 ^hijkl^	0.91 ± 0.03 ^kl^	0.00 ± 0.00 ^l^	5.35 ± 0.41 ^ghijkl^	5.65 ± 0.55 ^ghijkl^	3.78 ± 0.05 ^hijkl^	7.39 ± 0.59 ^efghijk^	4.92 ± 4.92 ^ghijkl^	8.34 ± 1.68 ^efghi^
T_7_ (Control)	12.25 ± 5.65 ^bcdef^	1.26 ± 0.12 ^jkl^	5.77 ± 0.33 ^ghijkl^	18.89 ± 1.92 ^ab^	21.49 ± 1.23 ^a^	17.74 ± 0.87 ^abc^	12.70 ± 0.33 ^bcde^	14.59 ± 4.40 ^defgh^	15.57 ± 3.59 ^abcd^
F-value									
Location	11.17								
Treatment	16.21								
Location* Treatment	0.83								

Values ± S.E. within a column followed by different letters differ significantly at *P* ≤ 0.05.

**Table 5:** Location-wise effect of bagging treatments on fruit borer infestation (%).

Treatment	Medziphema	Mohanpur	Sabour	Muzaffarpur	Ranchi	Ambikapur	Pantnagar	Neri	Gangian
T_1_	3.53 ± 1.23 ^jkl^	4.30 ± 0.16 ^ghij^	2.94 ± 1.35 ^hijk^	0.00 ± 0.00 ^l^	1.00 ± 0.17 ^jkl^	5.21 ± 4.81 ^fghij^	1.33 ± 0.94 ^jkl^	2.44 ± 2.44 ^ijkl^	0.01 ± 0.01 ^l^
T_2_	0.90 ± 0.51 ^jkl^	4.32 ± 0.13 ^ghij^	3.94 ± 1.55 ^hijk^	0.00 ± 0.00 ^l^	0.67 ± 0.00 ^kl^	4.94 ± 4.37 ^fghij^	1.34 ± 0.93 ^jkl^	2.56 ± 2.56 ^ijkl^	0.84 ± 0.18 ^jkl^
T_3_	0.94 ± 0.16 ^jkl^	3.91 ± 0.11 ^hijk^	6.71 ± 2.26 ^efghi^	1.92 ± 0.40 ^ijkl^	0.67 ± 0.00 ^kl^	4.21 ± 3.54 ^ghij^	1.45 ± 0.19 ^jkl^	2.43 ± 2.43 ^ijkl^	0.80 ± 0.62 ^jkl^
T_4_	1.13 ± 0.21 ^kl^	3.65 ± 0.12 ^hijk^	2.35 ± 0.92 ^ijkl^	0.00 ± 0.00 ^l^	1.17 ± 0.12 ^jkl^	5.12 ± 4.67 ^fghij^	1.34 ± 0.93 ^jkl^	2.00 ± 2.00 ^ijkl^	0.34 ± 0.24 ^kl^
T_5_	0.47 ± 0.14 ^kl^	4.44 ± 0.18 ^ghij^	2.68 ± 0.99 ^ijkl^	1.64 ± 0.53 ^ijkl^	1.00 ± 0.17 ^jkl^	5.97 ± 4.76 ^fghij^	1.78 ± 0.89 ^jkl^	2.33 ± 2.33 ^ijkl^	0.16 ± 0.16 ^kl^
T_6_	0.58 ± 0.18 ^jkl^	4.35 ± 0.14 ^ghij^	3.50 ± 1.07 ^hijk^	3.15 ± 0.33 ^hijk^	0.33 ± 0.00 ^kl^	5.72 ± 4.03 ^fghij^	1.18 ± 0.67 ^jkl^	2.44 ± 2.44 ^ijkl^	1.06 ± 0.43 ^jkl^
T_7_ (Control)	1.17 ± 0.26 ^a^	11.30 ± 0.21 ^bcde^	9.64 ± 0.34 ^cdef^	29.86 ± 2.07 ^ab^	15.70 ± 0.87 ^abcd^	16.97 ± 6.69 ^abc^	10.67 ± 0.04 ^bcdef^	11.29 ± 0.81 ^bcde^	0.01 ± 0.01 ^hijk^
F-value									
Location	5.88								
Treatment	62.60								
Location* Treatment	4.22								

Values ± S.E. within a column followed by different letters differ significantly at *P* ≤ 0.05.

Borer infestation was drastically suppressed by bagging at all locations, with infestation levels under bagged treatments typically remaining below 3%, irrespective of bag colour or timing. The significant location × treatment interaction observed for borer infestation reflects variability in regional pest pressure, but does not diminish the overall efficacy of bagging. Among the bagging treatments, early to mid-stage bagging (T4 and T5) tended to provide the most consistent reduction in cracking and sunburn, while all bagging treatments were equally effective in minimizing borer infestation.

### Effect of pre-harvest bagging on yield and fruit quality of litchi across locations

Fruit yield varied markedly among locations, with the highest yields recorded at Gangian, Pantnagar, Mohanpur, and Ambikapur, reflecting favourable growing conditions (**Table 6**). Across locations, bagged treatments consistently produced higher yields than the unbagged control, with yield advantages ranging from moderate to substantial depending on the environment. Pink non-woven polypropylene bags applied at 25–30 days after fruit set (T_5_ and T_6_) emerged as the most consistent yield-enhancing treatments, particularly at Gangian (≈81 kg tree^-^¹), Pantnagar (>64 kg tree^-^¹), Muzaffarpur (>60 kg tree^-^¹), and Mohanpur (>66 kg tree^-^¹). In contrast, the unbagged control exhibited significantly lower yields at most locations.

**Table 6:** Location-wise effect of bagging treatments on yield (kg/tree).

Treatment	Medziphema	Mohanpur	Sabour	Muzaffarpur	Ranchi	Ambikapur	Pantnagar	Neri	Gangian
T_1_	15.26 ± 1.32 ^klm^	60.20 ± 2.52 ^defg^	25.60 ± 1.03 ^l^	57.77 ± 0.55 fgh	40.41 ± 0.54 ^ij^	64.08 ± 3.09 ^cdef^	53.66 ± 5.54 ^gh^	38.94 ± 7.52 ^ijk^	69.07 ± 6.85 ^cde^
T_2_	15.56 ± 1.07 ^klm^	65.69 ± 2.87 ^cdef^	24.08 ± 0.94 ^lm^	58.82 ± 0.60 ^efg^	47.95 ± 0.63 ^hi^	63.84 ± 3.16 ^cdef^	57.06 ± 4.99 ^fgh^	41.33 ± 7.25 ^ij^	71.22 ± 7.42 ^bcde^
T_3_	16.40 ± 0.75 ^kl^	62.79 ± 2.47 ^def^	22.42 ± 0.86 ^m^	61.32 ± 0.64 ^def^	39.67 ± 0.54 ^ij^	66.84 ± 2.15 ^bcdef^	62.30 ± 4.78 ^def^	37.96 ± 7.21 ^jk^	81.69 ± 7.61 ^a^
T_4_	18.04 ± 0.40 ^jk^	59.82 ± 2.39 ^efg^	24.32 ± 0.91 ^lm^	57.47 ± 0.56 ^fgh^	45.17 ± 0.61 ^hi^	63.19 ± 2.17 ^cdef^	53.78 ± 4.83 ^gh^	42.09 ± 7.48 ^ij^	69.19 ± 6.94 ^cde^
T_5_	18.00 ± 1.15 j^k^	62.73 ± 2.55 ^def^	24.47 ± 0.92 ^lm^	59.27 ± 0.59 ^efg^	48.29 ± 0.65 ^hi^	63.78 ± 2.17 ^cdef^	55.25 ± 4.91 ^fgh^	44.57 ± 7.85 ^ij^	77.75 ± 8.12 ^ab^
T_6_	16.66 ± 0.80 ^kl^	66.49 ± 2.78 ^cdef^	26.35 ± 1.02 ^l^	60.73 ± 0.62 ^defg^	45.96 ± 0.62 ^hi^	64.21 ± 2.27 ^cdef^	64.21 ± 5.07 ^cdef^	38.81 ± 6.77 ^ijk^	81.24 ± 7.88 ^a^
T_7_ (Control)	11.44 ± 0.96 ^lm^	57.23 ± 2.31 ^fgh^	22.05 ± 0.87 ^m^	43.63 ± 0.46 ^ij^	31.72 ± 0.49 ^k^	58.69 ± 2.98 ^efg^	49.69 ± 4.56 ^hi^	38.23 ± 6.95 ^ijk^	56.33 ± 5.43 ^efgh^
F-value									
Location	215.45								
Treatment	4.60								
Location* Treatment	4.06								

Values ± S.E. within a column followed by different letters differ significantly at *P* ≤ 0.05.

Fruit weight followed trends similar to yield (**Table 7**). Across all centres, bagging significantly increased individual fruit weight compared with the control, with the most pronounced effects observed under mid- to late-stage bagging (T_3_–T_6_). The highest fruit weights were recorded at Pantnagar (up to 27.01 g), followed by Muzaffarpur, Sabour, and Gangian, whereas Medziphema and Ranchi recorded comparatively lower fruit weights across treatments.

**Table 7:** Location-wise effect of bagging treatments on fruit weight (g) of litchi.

Treatment	Medziphema	Mohanpur	Sabour	Muzaffarpur	Ranchi	Ambikapur	Pantnagar	Neri	Gangian
T_1_	14.89 ± 0.77 ^kl^	21.98 ± 0.26 ^de^	21.94 ± 0.26 ^de^	21.13 ± 0.26 ^efg^	18.65 ± 0.43 ^hij^	21.23 ± 0.06 ^defg^	27.01 ± 1.29 ^a^	20.37 ± 0.54 ^fg^	21.18 ± 1.09 ^defg^
T_2_	15.68 ± 0.39 ^jkl^	21.99 ± 0.57 ^de^	21.34 ± 0.35 ^defg^	22.37 ± 0.48 ^cd^	20.28 ± 0.89 ^fg^	21.62 ± 0.13 ^def^	24.52 ± 1.05 ^b^	19.78 ± 1.44 ^ghi^	20.79 ± 1.31 ^efg^
T_3_	16.39 ± 1.65 ^ijk^	21.10 ± 0.53 ^efg^	20.40 ± 0.06 ^fg^	22.91 ± 0.71 ^cd^	20.20 ± 0.46 ^fg^	22.54 ± 0.28 ^cd^	26.11 ± 0.73 ^ab^	18.96 ± 0.39 ^ghi^	21.71 ± 1.39 ^def^
T_4_	17.58 ± 1.76 ^hij^	21.20 ± 0.42 ^defg^	21.93 ± 0.13 ^de^	21.57 ± 0.30 ^def^	20.63 ± 0.58 ^efg^	20.38 ± 0.07 ^fg^	24.78 ± 0.96 ^b^	19.24 ± 1.22 ^ghi^	20.25 ± 0.91 ^fg^
T_5_	18.60 ± 1.08 ^hij^	21.74 ± 0.58 ^def^	22.76 ± 0.43 ^cd^	22.12 ± 0.42 ^cde^	21.79 ± 0.75 ^def^	20.78 ± 0.05 ^efg^	25.01 ± 0.91 ^b^	21.27 ± 1.47 ^defg^	21.70 ± 1.33 ^def^
T_6_	16.77 ± 0.55 ^ij^	21.47 ± 0.51 ^def^	23.61 ± 0.30 ^bc^	22.40 ± 0.36 ^cd^	21.69 ± 0.60 ^def^	21.79 ± 0.22 ^def^	26.07 ± 0.98 ^ab^	19.71 ± 0.78 ^ghi^	22.90 ± 1.19 ^cd^
T_7_ (Control)	11.84 ± 1.41 ^l^	20.25 ± 0.30 ^fg^	20.00 ± 0.09 ^fg^	19.35 ± 0.28 ^ghi^	16.96 ± 0.25 ^ij^	19.88 ± 0.28 ^ghi^	22.92 ± 0.83 ^cd^	17.36 ± 1.36 ^hij^	18.53 ± 0.83 ^hij^
F-value									
Location	42.50								
Treatment	3.27								
Location* Treatment	2.32								

Values ± S.E. within a column followed by different letters differ significantly at *P* ≤ 0.05.

Anthocyanin accumulation, a key determinant of pericarp colour, showed significant enhancement under bagging treatments (**Table 8**). The response was highly location-dependent, with the highest anthocyanin content recorded at Ranchi, where pink bagging treatments (T_4_–T_6_) consistently produced superior pigmentation. At Muzaffarpur and Sabour, late-stage bagging resulted in higher anthocyanin levels compared with early bagging and the control. In contrast, Medziphema and Pantnagar showed comparatively moderate responses, reflecting the strong influence of regional climatic conditions on pigment biosynthesis.

**Table 8:** Location-wise effect of bagging treatments on anthocyanin content (mg/100mg).

Treatment	Medziphema	Mohanpur	Sabour	Muzaffarpur	Ranchi	Ambikapur	Pantnagar	Neri	Gangian
T_1_	18.33 ± 0.41 ^lmn^	23.23 ± 0.17 ^ghijkl^	74.19 ± 0.00 ^ghijkl^	35.92 ± 2.37 ^def^	67.71 ± 3.78 ^b^	30.23 ± 3.43 ^efg^	26.20 ± 0.11 ^fghi^	30.38 ± 1.23 ^efg^	22.45 ± 1.64 ^ghijkl^
T_2_	16.15 ± 1.69 ^lmn^	22.60 ± 0.86 ^ghijkl^	23.42 ± 0.12 ^ghijkl^	34.09 ± 1.84 ^ef^	69.26 ± 2.78 ^b^	28.05 ± 2.46 ^fgh^	24.03 ± 0.82 ^fghijk^	32.24 ± 1.06 ^ef^	23.03 ± 1.19 ^ghijkl^
T_3_	17.80 ± 1.01 ^lmn^	23.07 ± 0.20 ^ghijkl^	23.21 ± 0.15 ^ghijkl^	36.85 ± 2.65 ^cde^	67.43 ± 2.54 ^b^	29.58 ± 2.00 ^fgh^	23.77 ± 1.48 ^ghijkl^	31.10 ± 2.03 ^efg^	24.20 ± 1.28 ^fghijk^
T_4_	16.26 ± 2.22 ^lmn^	22.83 ± 0.23 ^ghijkl^	22.87 ± 0.21 ^fghi^	34.12 ± 1.67 ^ef^	73.48 ± 0.00 ^a^	27.65 ± 1.23 ^fghi^	22.63 ± 0.64 ^ghijkl^	29.05 ± 0.95 ^fgh^	24.40 ± 0.90 ^fghijk^
T_5_	17.22 ± 2.95 ^lmn^	23.24 ± 0.18 ^ghijkl^	26.27 ± 0.17 ^fghijk^	36.41 ± 1.07 ^cde^	75.80 ± 0.00 ^a^	27.55 ± 2.08 ^fghi^	24.16 ± 0.35 ^fghijk^	31.41 ± 0.88 ^efg^	24.05 ± 2.14 fghijk
T_6_	17.19 ± 2.37 ^klmn^	22.59 ± 0.29 ^ghijkl^	23.96 ± 0.19 ^ghijkl^	37.82 ± 0.92 ^cd^	74.02 ± 0.00 ^a^	26.40 ± 1.89 ^fghi^	26.39 ± 0.09 ^fghi^	32.82 ± 1.09 ^ef^	23.40 ± 1.12 ^ghijkl^
T_7_ (Control)	18.36 ± 2.97 ^n^	19.73 ± 0.25 ^jklmn^	23.16 ± 0.15 ^hijkl^	25.47 ± 0.14 ^fghij^	67.71 ± 3.78 ^a^	21.33 ± 0.45 ^hijkl^	23.35 ± 0.33 ^ghijkl^	20.47 ± 0.12 ^ijkl^	22.45 ± 1.64 ^klmn^
F-value									
Location	6.82								
Treatment	12.51								
Location* Treatment	3.91								

Values ± S.E. within a column followed by different letters differ significantly at *P* ≤ 0.05.

Bagging exerted a strong influence on total soluble solids (TSS), with significant effects of location, treatment, and their interaction (**Table 9**). Across locations, bagged fruits generally recorded higher TSS than unbagged fruits, particularly under T_3_–T_6_. TSS enhancement was most pronounced at Sabour, Ambikapur, Ranchi, and Muzaffarpur, where late-stage bagging resulted in the highest sugar accumulation. Although differences among treatments were smaller at Pantnagar and Medziphema, bagged fruits consistently maintained higher TSS compared with the control, indicating a stabilizing effect of bagging on sugar metabolism.

**Table 9:** Location-wise effect of bagging treatments on TSS (°B).

Treatment	Medziphema	Mohanpur	Sabour	Muzaffarpur	Ranchi	Ambikapur	Pantnagar	Neri	Gangian
T_1_	16.61 ± 1.34 ^hij^	19.43 ± 0.04 ^defgh^	18.53 ± 0.60 ^efghi^	19.53 ± 0.26 ^cdefg^	19.94 ± 0.53 ^cdefg^	19.53 ± 0.02 ^cdefg^	18.00 ± 0.03 ^fghi^	16.86 ± 0.58 ^ghi^	20.32 ± 0.86 ^bcde^
T_2_	18.07 ± 1.00 ^fghi^	19.41 ± 0.03 ^defgh^	19.86 ± 0.24 ^cdefg^	19.08 ± 0.39 ^efgh^	20.66 ± 0.43 ^abcd^	20.32 ± 0.06 ^bcde^	18.15 ± 0.10 ^fghi^	17.95 ± 0.94 ^fghi^	19.30 ± 0.53 ^defgh^
T_3_	13.52 ± 4.10 _j_	19.28 ± 0.06 ^defgh^	20.43 ± 0.17 ^bcde^	19.70 ± 0.25 ^cdefg^	20.11 ± 0.50 ^bcdef^	21.15 ± 0.04 ^ab^	18.23 ± 0.06 ^fghi^	17.82 ± 0.66 ^fghi^	19.70 ± 0.30 ^cdefg^
T_4_	17.87 ± 1.13 ^fghi^	19.35 ± 0.03 ^defgh^	19.55 ± 0.63 ^cdefg^	19.07 ± 0.52 ^efgh^	20.15 ± 0.41 ^bcdef^	19.22 ± 0.04 ^defgh^	18.46 ± 0.08 ^efghi^	17.14 ± 1.10 ^ghi^	20.13 ± 0.41 ^bcdef^
T_5_	17.06 ± 0.47 ^ghi^	19.58 ± 0.02 ^cdefg^	20.65 ± 0.43 ^abcd^	19.47 ± 0.32 ^defgh^	20.08 ± 0.38 ^bcdef^	19.96 ± 0.18 ^cdefg^	18.51 ± 0.09 ^efghi^	18.90 ± 0.78 ^efghi^	19.94 ± 0.15 ^cdefg^
T_6_	17.20 ± 0.80 ^ghi^	19.60 ± 0.02 ^cdefg^	21.50 ± 0.30 ^a^	19.50 ± 0.28 ^cdefg^	19.26 ± 0.57 ^defgh^	20.81 ± 0.27 ^abc^	18.09 ± 0.12 ^fghi^	17.99 ± 0.47 ^fghi^	19.95 ± 0.42 ^cdefg^
T_7_ (Control)	14.53 ± 0.68 ^ij^	18.91 ± 0.04 ^efghi^	18.72 ± 0.33 ^efghi^	19.40 ± 0.30 ^defgh^	19.52 ± 0.30 ^cdefg^	18.60 ± 0.18 ^efghi^	17.74 ± 0.30 ^fghi^	17.37 ± 0.61 ^ghi^	18.82 ± 0.55 ^efghi^
F-value									
Location	78.9								
Treatment	18.83								
Location* Treatment	10.95								

Values ± S.E. within a column followed by different letters differ significantly at *P* ≤ 0.05.

Fruit acidity was also significantly affected by bagging, with a strong location × treatment interaction (**Table 10**). In most locations, bagging reduced or moderated acidity relative to the control, resulting in a more favourable sugar–acid balance. The lowest acidity levels were generally observed under mid- to late-stage bagging (T_3_–T_6_), particularly at Pantnagar, Ambikapur, and Neri, whereas the control frequently exhibited higher acidity.

**Table 10:** Location-wise effect of bagging treatments on acidity (%).

Treatment	Medziphema	Mohanpur	Sabour	Muzaffarpur	Ranchi	Ambikapur	Pantnagar	Neri	Gangian
T_1_	0.34 ± 0.07 ^ghij^	0.59 ± 0.01 ^ab^	0.42 ± 0.02 ^efgh^	0.52 ± 0.04 ^bcde^	0.15 ± 0.02 ^m^	0.37 ± 0.02^ghi^	0.30 ± 0.01^ijk^	0.47 ± 0.08 ^defg^	0.45 ± 0.09 ^defg^
T_2_	0.32 ± 0.07 ^hij^	0.58 ± 0.01 ^abc^	0.36 ± 0.03 ^ghij^	0.61 ± 0.05 ^a^	0.14 ± 0.01 ^m^	0.31 ± 0.03 ^hij^	0.26 ± 0.01 ^kl^	0.40 ± 0.02 ^fghi^	0.50 ± 0.11 ^cdef^
T_3_	0.40 ± 0.03 ^fghi^	0.58 ± 0.02 ^abc^	0.43 ± 0.01 ^efgh^	0.51 ± 0.02 ^bcdef^	0.16 ± 0.02 ^m^	0.38 ± 0.01^ghi^	0.23 ± 0.01 ^l^	0.52 ± 0.03 ^bcde^	0.41 ± 0.03 ^efgh^
T_4_	0.28 ± 0.05 ^ijk^	0.60 ± 0.01 ^a^	0.40 ± 0.01 ^fghi^	0.55 ± 0.03 ^abcd^	0.13 ± 0.01 ^m^	0.35 ± 0.01 ^ghij^	0.29 ± 0.01 ^ijk^	0.51 ± 0.03 ^bcdef^	0.42 ± 0.11 ^efgh^
T_5_	0.31 ± 0.09 ^hij^	0.58 ± 0.01 ^abc^	0.43 ± 0.01 ^efgh^	0.51 ± 0.02 ^bcdef^	0.14 ± 0.01 ^m^	0.38 ± 0.03 ^ghi^	0.26 ± 0.01 ^kl^	0.43 ± 0.01 ^efgh^	0.39 ± 0.04 ^fghi^
T_6_	0.29 ± 0.06 ^ijk^	0.61 ± 0.01 ^a^	0.43 ± 0.03 ^efgh^	0.58 ± 0.02 ^abc^	0.16 ± 0.01 ^m^	0.38 ± 0.01 ^ghi^	0.23 ± 0.01 ^l^	0.56 ± 0.01 ^abcd^	0.44 ± 0.08 ^defgh^
T_7_ (Control)	0.54 ± 0.04 ^bcd^	0.58 ± 0.01 ^abc^	0.49 ± 0.03 ^cdefg^	0.37 ± 0.01 ^ghi^	0.13 ± 0.01 ^m^	0.44 ± 0.03 ^defgh^	0.32 ± 0.01 ^hij^	0.58 ± 0.03 ^abc^	0.53 ± 0.11 ^bcde^
F-value									
Location	217.89								
Treatment	13.62								
Location* Treatment	19.43								

Values ± S.E. within a column followed by different letters differ significantly at *P* ≤ 0.05.

**Figure 2:**
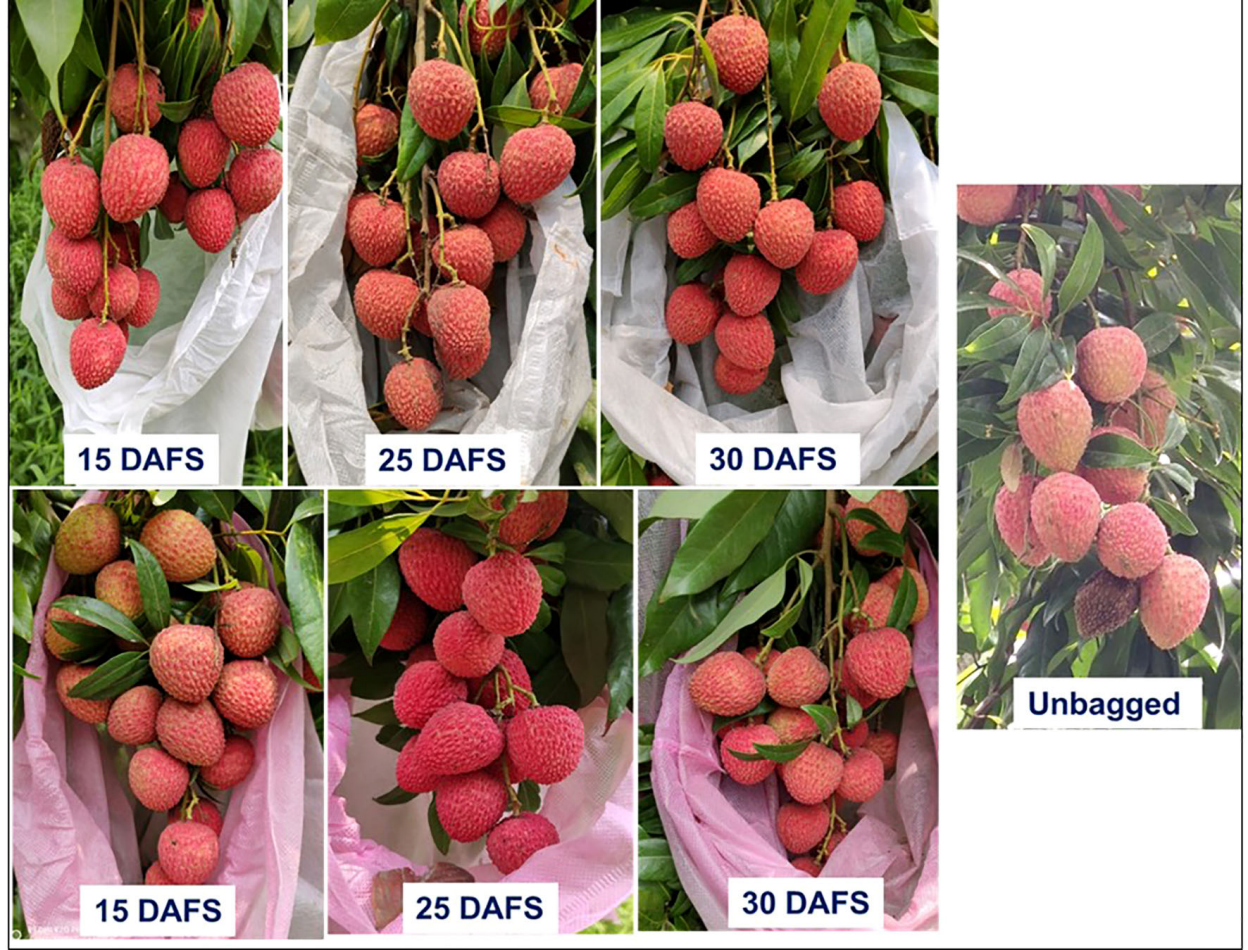
Effect of pre-harvest bunch bagging on fruit appearance and colour development in litchi at Muzaffarpur, Bihar. Representative fruit bunches bagged with non-woven polypropylene bags at 15, 25 and 30 days after fruit set (DAFS) using white and pink bags, compared with unbagged control fruits exposed to natural environmental conditions.

Representative images illustrating the effect of bagging material and timing of application on fruit appearance are presented in **Figure 2**. Across both bagging materials, bagging at 25 and 30 days after fruit set (DAFS) resulted in visibly superior fruit colour development, uniform pericarp texture, and reduced surface blemishes compared with early bagging at 15 DAFS and the unbagged control. Fruits bagged at 15 DAFS exhibited comparatively lighter and less uniform red coloration. In contrast, bagging at 25 DAFS produced fruits with intense, uniform red pericarp, smooth surface texture, and better overall visual appeal, indicating an optimal balance between protection and physiological colour development. Bagging at 30 DAFS also enhanced colour and appearance; however, fruits were slightly less uniform than those bagged at 25 DAFS. The unbagged fruits showed uneven coloration, surface browning, and visible blemishes, reflecting greater exposure to solar radiation and environmental stresses during the critical maturation phase. Differences between bagged and unbagged fruits clearly demonstrate the role of bagging in improving pericarp colour and marketable appearance.

### Multivariate clustering of bagging treatments based on yield, fruit quality, and damage-related traits

**Figure 3:**
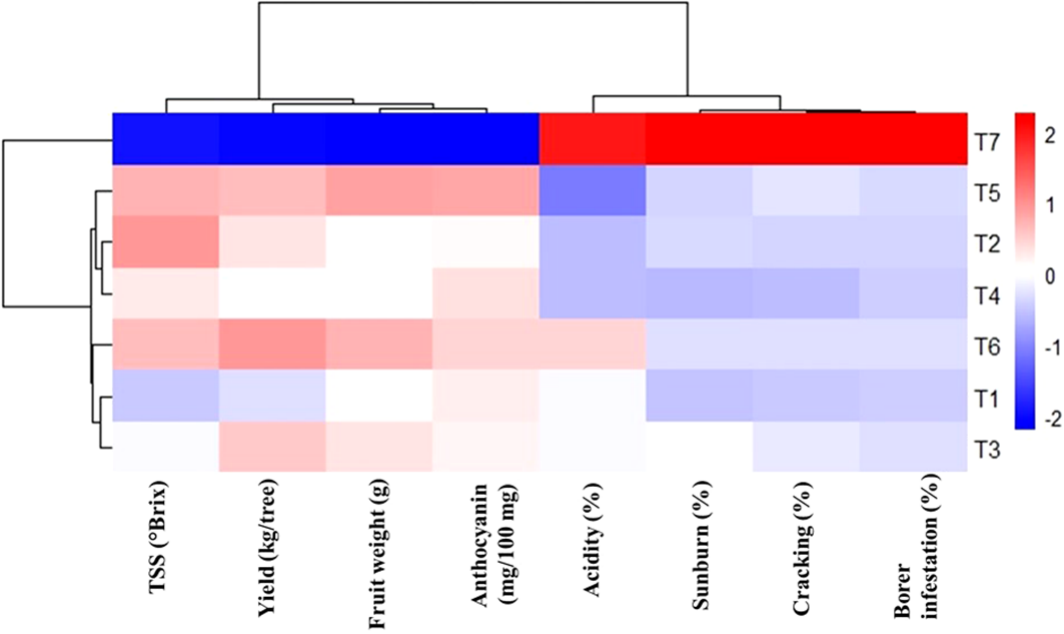
Hierarchical clustering heatmap showing standardized responses of litchi bagging treatments based on yield, fruit quality, and damage-related traits. The color scale indicates relative trait values, with red denoting higher standardized values and blue denoting lower standardized values. Higher values (red) for yield and quality attributes indicate superior fruit performance, whereas lower values (blue) for sunburn, cracking, borer infestation, and acidity indicate effective mitigation of stress and damage.

Hierarchical clustering heatmap analysis based on standardized trait values revealed a clear separation of bagging treatments into distinct performance groups (**Figure 3**). The treatments clustered primarily according to their combined effects on yield, fruit quality attributes, and stress-related parameters, highlighting the multidimensional impact of pre-harvest bagging. The unbagged control (T_7_) formed a distinct and isolated cluster, characterized by high positive standardized values for sunburn, fruit cracking, borer infestation, and acidity, and negative associations with fruit weight, yield, TSS, and anthocyanin content. This clustering pattern confirms the consistently inferior performance of unbagged fruits across locations and underscores their greater susceptibility to biotic and abiotic stresses.

In contrast, bagging treatments were grouped into two major clusters, reflecting intermediate and superior performance levels. Treatments T_5_ and T_6_ clustered closely together and were strongly associated with higher fruit yield, greater fruit weight, enhanced TSS and anthocyanin accumulation, and lower incidence of cracking, sunburn, and borer infestation. This cluster represents the most desirable multi-trait performance profile, indicating the robustness of pink non-woven polypropylene bagging applied at 25–30 days after fruit set. Treatments T_1_–T_4_ formed an intermediate cluster, exhibiting moderate improvements in yield and fruit quality relative to the control but comparatively weaker performance than T_5_ and T_6_. Within this group, early-stage bagging treatments showed partial mitigation of stress-related traits but were less effective in maximizing biochemical quality attributes.

### Multivariate discrimination of bagging treatments using principal component analysis

**Figure 4:**
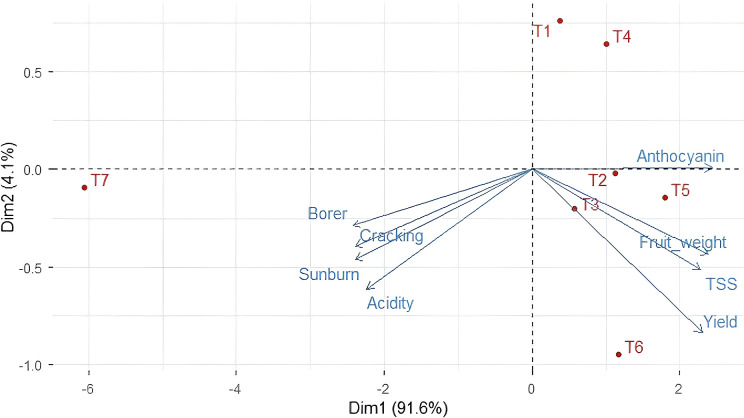
Principal component analysis (PCA) biplot showing relationships among bagging treatments and yield, fruit quality, and damage-related traits of litchi based on standardized data. Vectors indicate trait loadings, while points represent bagging treatments.

Principal component analysis (PCA) based on standardized yield, fruit quality, and damage-related traits revealed a clear multivariate separation of bagging treatments (**Figure 4**). The first principal component (PC1) explained 91.6% of the total variation, while the second principal component (PC2) accounted for an additional 4.1%, together capturing the major sources of variability among treatments. PC1 primarily reflected a strong contrast between productive and quality-enhancing traits and stress- and damage-associated traits. Positive loadings on PC1 were dominated by yield, fruit weight, total soluble solids (TSS), and anthocyanin content, whereas negative loadings were associated with sunburn, fruit cracking, borer infestation, and acidity, indicating an inverse relationship between fruit quality/yield attributes and damage-related parameters.

Bagging treatments T_5_ and T_6_ clustered distinctly on the positive side of PC1, showing strong alignment with yield and quality-related vectors, suggesting their superior and stable performance across locations. Treatments T_2_ and T_3_ occupied intermediate positions, indicating moderate improvement in fruit quality and yield attributes relative to the control. In contrast, the unbagged control (T_7_) was clearly separated along the negative PC1 axis and closely associated with sunburn, cracking, and borer infestation, highlighting its susceptibility to both abiotic and biotic stresses. PC2 contributed to secondary discrimination among treatments, mainly differentiating early-stage bagging treatments from mid- and late-stage applications, suggesting treatment-specific variation related to the timing of bagging rather than overall performance.

## Discussion

In this multi-location evaluation of pre-harvest bagging in litchi (*Litchi chinensis* Sonn.), we demonstrate that bagging markedly mitigates fruit cracking, sunburn, and borer infestation while enhancing fruit yield and quality across diverse agro-climatic environments. These outcomes align with recent studies emphasizing the role of microclimate modification and physical protection in improving fruit physiology and marketability (Deepti et al., 2025; Mustafa et al., 2025; Nadeem et al., 2022).

### Pre-harvest bagging reduces fruit cracking, sunburn, and borer infestation

Fruit cracking, sunburn, and borer infestation were significantly reduced by pre-harvest bagging across all locations, with exposed fruits (T_7_) consistently exhibiting the highest damage levels (**Tables 3**–**5**). These disorders are closely linked to abrupt fluctuations in temperature, solar radiation, and moisture during rapid fruit expansion (Huang, 2005). In the present study, bagging restricted fruit cracking and sunburn largely to ≤5–7% across environments, confirming the effectiveness of microclimate moderation through reduced radiation load and stabilized humidity, as previously reported in pomegranate (Habibli et al., 2025). Cracking and sunburn were particularly severe in the unbagged control at Ranchi, Ambikapur, Muzaffarpur, Pantnagar, and Gangian, reflecting greater exposure to thermal and radiation stress under open-canopy conditions. In contrast, cracking and sunburn were almost eliminated at Sabour and Mohanpur under bagged treatments, highlighting the synergistic effect of bagging under humid subtropical conditions (**Table 3**, **4**). Borer infestation was drastically suppressed by bagging at all locations, typically remaining below 3%, irrespective of bag colour or timing (**Table 5**). This reflects the role of bagging as a physical barrier preventing oviposition, consistent with earlier observations that female litchi fruit borers preferentially lay eggs on exposed mature fruits (Meng et al., 2018). Although a significant location × treatment interaction was observed for borer infestation, indicating variation in regional pest pressure, the consistently low infestation under bagged treatments underscores the robustness of this non-chemical intervention (Kumar et al., 2014b; Ramakrishnaiah et al., 2017).

### Effects of bagging on yield and fruit weight

Fruit yield and individual fruit weight varied significantly among locations but were consistently enhanced by bagging relative to the unbagged control (**Table 6**, **7**). The highest and most stable yield responses were obtained with pink non-woven polypropylene bags applied at 25–30 days after fruit set (T_5_ and T_6_), particularly at Gangian, Pantnagar, Muzaffarpur, and Mohanpur. These gains likely result from reduced pre-harvest losses and improved fruit retention under protected microclimatic conditions, as reported in peach and date palm (Alebidi et al., 2024; Kim et al., 2003). Fruit weight followed trends similar to yield, with mid- to late-stage bagging (T_3_–T_6_) outperforming early bagging and the control. Late-stage protection appears particularly important during rapid aril expansion, supporting earlier reports that duration of protection, rather than bag type alone, governs fruit size responses (Kim et al., 2003). Lower fruit weights at Medziphema and Ranchi across treatments further highlight the strong influence of regional climate on size potential.

### Influence of bagging on pericarp coloration and fruit biochemical quality

Anthocyanin accumulation, a key determinant of litchi pericarp colour, was strongly enhanced by bagging, particularly with pink bags applied at 25–30 DAFS (**Table 8**; **Figure 2**). The strongest responses were observed at Ranchi and Muzaffarpur, while more moderate effects occurred at Medziphema and Pantnagar, reflecting climatic modulation of pigment biosynthesis. Anthocyanin synthesis in litchi is sensitive to light and temperature (Zhang et al., 2004), and moderated exposure under bagging likely stabilizes flavonoid pathways, as reported in apple and kiwifruit (Liu et al., 2013; Sharma et al., 2013; Yu et al., 2024). Visual improvements in colour uniformity and surface quality under mid-stage bagging further confirm these biochemical responses (**Figure 2**). Bagging significantly improved fruit quality, including TSS, and sugar–acid balance (**Table 10**). Across locations, bagged fruits generally exhibited higher TSS and moderated acidity compared with the unbagged control, indicating improved physiological maturity. These responses are consistent with studies demonstrating enhanced carbohydrate accumulation and acid metabolism under modified fruit microclimates (Ni et al., 2011; Sohag et al., 2023).

Non-woven bags have recently emerged as an alternative to conventional plastic bags in horticultural production. With increasing global concern over plastic pollution, agricultural systems are actively exploring greener options such as non-woven materials (Kopitar et al., 2022). These bags are reported to be reusable, recyclable, and biodegradable, with complete biodegradation occurring within 1–6 months depending on their material composition. Unlike plastic bags, non-woven materials permit free air and moisture exchange around the fruit surface, thereby improving the fruit microenvironment (Deepti et al., 2025). Owing to these attributes, non-woven bags are considered a relatively more sustainable option for agricultural applications and represent a greener alternative to plastic-based bagging systems (Kalukhe et al., 2024).

## Conclusion

Collectively, the present study establishes pre-harvest bunch bagging as a robust, eco-safe, and location-resilient management strategy for litchi production across diverse agro-climatic regions. In particular, the use of pink non-woven polypropylene bags applied at 25–30 days after fruit set consistently delivered superior outcomes by markedly reducing physiological disorders such as fruit cracking and sunburn, suppressing borer infestation, and enhancing yield and key fruit quality attributes. The concurrent improvement in productivity, biochemical quality, and visual appeal of fruits highlights the multifaceted benefits of bagging and its potential to substantially reduce dependence on chemical pesticides during the critical fruit development and maturation stages. The strong and consistent treatment responses observed across nine contrasting locations underscore the wide adaptability and scalability of this intervention. These findings support the integration of optimized pre-harvest bagging practices into good agricultural practices for litchi, particularly in the context of sustainable and export-oriented production systems. Adoption of this approach can contribute meaningfully to improving market competitiveness, environmental sustainability, and resilience of litchi cultivation under variable climatic conditions.

## Data Availability

The original contributions presented in the study are included in the article/supplementary material. Further inquiries can be directed to the corresponding authors.
